# The Sanitary Regulations of Antient Rome
*T. Livii Patavini Hist. Ed. Drakenborchii.Galeni, Opera. Edit. Kühn.Taciti, Annal.Suetonius, Vitæ Imperator.Gibbon, Decline and Fall of the Roman Empire. London.C. Plinii, Cæc. Secundi Epistolæ. Weise.D'Arnay's Private Life of the Romans. Edinburgh.Smith (Dr. W.) Dictionary of Greek and Roman Antiquities.Smith (Dr. W.) Greek and Roman Biography.Gell's Topography of Rome.


**Published:** 1855-09

**Authors:** Alfred Haviland


### REVIEWS.

### (CRITICAL, HISTORICAL, AND ANALYTICAL.)

### THE SANITARY REGULATIONS OF ANTIENT ROME.*

PART II.

*Remarkable Epidemics.* The object of this Journal is to lay before the public, the legislature, and the medical profession, the various social and other causes of disease, and to instil into the minds of all, both the necessity of preventing what they cannot cure, and of alleviating what they cannot prevent. A detailed history of the plagues and pestilences, which from the earliest periods have infested Rome, would perhaps convey but little information so far as sanitation is concerned, although a classification of the various causes of these epidemics will help us to unravel the mysteries of these insidious foes. Livy and other historians are found relating what, at the time of the raging of a pestilence, was considered to be the *origo mali.* Scattered and insufficient as this part of their information nearly always is, we will attempt to string together what we have gleaned on this point, and thus save our readers the trouble which such a task necessarily entails. For the most part we find that the exciting causes were malaria from the plains, putrid emanations from dead bodies either within or without the city, concentrated animal exhalations from close confinement, insufficient water supply, defective drainage, and certain conditions of the atmosphere conducive to an universal epidemic. Among the predisposing causes may be mentioned famine, depression of the public mind either from defeat, sedition, or a superstitious dread arising from a combination of political disasters, and unusual meteoric phenomena, luxury, debauchery, and unnatural habits. The pestilence that broke out B.C. 640, in the reign of Tullus Hostilius, was preceded by a shower of stones on Mount Albanus, accompanied by rumbling noises and followed by a thunderstorm. The next, B.C. 462, was of a very severe character, carried off 100,000 inhabitants, and was attributed to the unhealthiness of the season: the people were terrified by their enemies, the Æquians, and sought refuge within the walls of the city, where the intolerable stench from the cattle affected the citizen, and the close confinement the countryman. Added to all this, the minds of the people were in an ill condition, prostrate

---

* T. LIVII PATAVINI Hist. Ed. Drakenborchii.
GALENI, Opera. Edit. Kühn.
TACITI, Annal.
SUETONIUS, Vitæ Imperator.
GIBBON, Decline and Fall of the Roman Empire. London.
C. PLINII, Cæc. Secundi Epistolæ. Weise.
D'ARNAY'S Private Life of the Romans. Edinburgh.
SMITH (Dr. W.) Dictionary of Greek and Roman Antiquities.
——————— Greek and Roman Biography.
GELL'S Topography of Rome.

and anxious as they were in consequence of the foe without and the pestilence within. In B.C. 451, murrain and pestilence succeeded to famine. B.C. 433-4, were unhealthy years, the latter more so than the former, during which earthquakes were so prevalent as to destroy whole villages; the city was vexed also by sedition, which with the bad news from the provinces, and the present distemper, combined to unhinge the public mind. B.C. 430, scarcity and disease again went hand in hand. B.C. 427, a drought was the cause of an epidemic; the scarcity of water drove the cattle to the hot springs, after which a disease, described by Livy as scabies, attacked them, and then by contagion those who attended them; the disease at last became general. B.C. 409, famine succeeded pestilence, instead of being the precursor as usual. The year B.C. 399 was remarkable for a severe winter, which rendered the Tiber unnavigable, and thus checked the necessary importations; the summer was hot and unhealthy, and as Livy remarks, this great change from cold to heat was in all probability the cause of the pestilence which then raged so fearfully; in addition, however, to a ravaging epidemic, the Romans had to contend with their enemies the Veii, and their superstitious alarm created by the rising of the waters of Lake Albanus to an extraordinary height. Another great plague broke out during the year B.C. 365-4, to which Camillus became a victim. The years B.C. 380, 344, 311, and 293, were distinguished by unusual epidemics. The thunderstorm in B.C. 295 was terrific, the bolts killing the soldiers of the Prætorian guards, coincident with which was pestilence. A.D. 78, a most awful visitation took place at Rome; 10,000 people died daily. A.D. 167, the same disease ravaged the Roman empire at the time when Galen was practising in the city. A.D. 252, there was also a long continued devastation from plague which spread over large tracts of country, and in its course visited Rome; it prevailed for fifteen years.

Thus will the reader of Roman history find that those people were often sorely tried by a combination of miseries; for amidst war, famine, sedition, and their superstitious dread of the meteoric phenomena, which sometimes by their grandeur overawed and depressed them, thus rendering them incapable of resisting their insidious and ever ready enemy, *malaria,* they had to contend with their neighbours in many a sanguinary struggle; and often the stench from the dead bodies outside the walls lent its aid to the besiegers.

### II.—Regulations for Personal Cleanliness, Supply of Water, Aqueducts, Baths, etc.

*The Aqueducts of Rome.* In consequence of the badness and deficiency of water, this invaluable prophylactic was obliged to be brought to the city by means of aqueducts. There were several of these magnificent structures, according to some as many as twenty, whilst others reduce the number to fourteen. One brought water upwards of sixty miles: rocks were hewn through, mountains tunneled, and valleys arched for the purpose of carrying out this gigantic design. Some of the arches were 109 feet high. The date of the first aqueduct, according to Frontinus, is assigned to the year A.U.C. 441. The water thus brought into the city was received into enormous reservoirs called *Castella*, which were divided into *privata* and *publica*. The former were intended to provide private houses with water, which was raised into a leaden cistern called *castellum domesticum*. The *Castella publica* supplied the Prætorian camp, the ponds and fountains, the circus, naumachiæ, amphitheatres, baths, and certain trades, such as fullers, dyers, and tanners, etc. (Smith.) Government was always very jealous in the preservation of the water, and heavy penalties were inflicted on those who either wasted or purloined it; a vigilant police was appointed to superintend the works. The curator and ædiles were appointed curators of these buildings under the republic. Augustus, however, established the office of *curator* or *præfectus aquarum*, who had an efficient staff under him; for instance, in the time of Trajan, a body of 460 slaves were constantly employed under the orders of the *curatores aquarum*. Some had to take care of the pipes, others the reservoirs, some were masons and paviors, and others water carriers. The system seemed complete, and connected as it was with the magnificent baths, we may look upon the supply of water in ancient Rome as the most perfect piece of sanitary provision that the history of this city contains. It employed thousands in the constructing of these buildings, hundreds were constantly engaged about them when completed, it provided thousands with pure water for washing and drinking daily, it afforded amusement after the toils of war or the fatigues of business; it flushed the sewers, and thus purified their houses and streets. With such an amount of physical cleansing, a wholesome moral effect must have gone hand in hand, and thus not only have raised the standard of the bodily health, but have most efficiently contributed to the psychical health of the community at large.

*Baths.* The early Romans, whilst enjoying simplicity in their manners and tastes, contented themselves with washing their legs, arms, and faces once a day, and using a general bath once a week only. During the republic, public baths were erected and used by the people with benefit; they were dark, however, and far from commodious; their simplicity of structure declined as wealth, and its child, the love of luxury began to shed its seductive smile upon the conquering Romans, until, under the emperors, the public *thermæ* were the most magnificent structures in the city, and adapted for every kind of enjoyment. At times, men and women were allowed to bathe promiscuously, although subsequently the demoralising practice was put a stop to by Hadrian and Alexander Severus. Then later still, as the fall of Rome loomed in the distance, invited by the soft luxuriance and enervating effeminacy of her people, we find the bath resorted to for the sake of luxury, instead of cleanliness, several times a day. Before meals and after them did the gluttonous gourmand bathe, in the hope of facilitating digestion, thus affording him a chance for an extra meal. Some even had their meals in the bath, and, in some instances, substituted the milk of asses for water. Alexander Severus, at a time when bathing had become quite a mania, gratified the people by opening the public baths before daybreak, at which time lamps were allowed to the bathers.

Such is an outline of the progress of the bath at Rome. We will now give a few details in regard to the regulations, &c., of these public institutions. The baths of Rome were far different from our public baths and wash-houses; they were not only regarded as necessary to cleanliness and health, but as pre-eminently useful in bringing men together, who, when thus congregated, amused each other by competition in feats of strength, gossip, literary discussions; in fact, by every means calculated to dissipate time and relax the mind by diverting the thoughts into a train different from that in which they had been during business hours. The mind is not relaxed by thinking or acting upon nothing; it, as it were, feeds upon itself. Some men certainly feel a sort of enjoyment in the vacuity of mind, like the country clown who thought the height of ease was to think of nothing during the sermon of his pastor. Nothing, however, tends so much to maintain the *mens sana* as keeping it employed. It is that dreadful sense of vacuity which leads men, intolerant of it from constant mental activity, to commit excesses and enter into unworthy society in order to rid themselves of *ennui*. When many are idle, there will be many overtaxed, either bodily or mentally. The Romans seemed instinctively to be aware of this fact, and many were the means to which they resorted in order to ameliorate the evil. At the baths the idler amused the hard-worker, who was glad to escape from the drudgery of business and be refreshed by the variety of characters with which the baths always swarmed.

The business of the Roman was generally finished by the middle of the day; he then took exercise in the campus Martius or some other place devoted to this purpose.

"Post decisa negotia campo."*

There he exerted himself well with the ball, ran, leapt, or, if a literary character, read aloud; after which he was stripped, and all sweat and dirt was rubbed off him. He then used the warm and cold baths consecutively, after emerging from which he was again well rubbed with rough towels, anointed and perfumed, during which he gossiped on the topics of the day until supper time, which they very sensibly made their chief meal: it corresponded to our late dinner. A meal taken at such an hour did not interfere with business, and was therefore less liable to interruption. The cares of the day being to a certain extent over, the mind became

* Hor. Ep. i, 7, 54.

easy, and thus admitted of a more vigorous digestion. All this exercise and bathing must have had a most decided effect upon the health of the Roman people, and have rendered them less liable to be affected by the unhealthiness of the surrounding country. The regularity of the operation was not without its good influence. The people were not allowed to bathe, in the public baths, at least, until about two or three o'clock in the afternoon, nor after dusk. Of course in such large establishments, where so many resorted, a large quantity of water was required, which took some time to heat. The Romans began with the hot or tepid bath, and ended with the *frigidarium*. When the *caldarium* was ready, a bell was rung to announce it. The sick were forbidden to bathe, except with their physician's leave. Surely those in authority had a due regard for the public health when, as Hadrian did, they guided their people towards its attainment by so many wise sanitary regulations. The baths were closed about four; under some of his successors, however, they were continued open until a later hour, a hundredfold the price, namely, a hundred *quadrantes*, was demanded. During the time of Nero the baths were hot at twelve. After the bath a state of repose was required, and this was attained by reclining on couches during their supper, for which the higher classes in Roman society always dressed as we do for dinner: thus it is that customs are derived one from the other. Celsus did not approve of the regular use of the bath; in fact, he observed truly that the life of a healthy man ought to be as varied as possible, whether it be in bathing, exercise, food, or rest. Antonius Musa is said to have been the first to propose the use of the cold bath as a medical agent, although its use must have been as old as bathing itself: it, however, became fashionable after this physician had advised Augustus to take it, and the treatment proved successful. This *mode*, however, had but a short life, for Marcellus died from the effect of using it injudiciously. For this service to Augustus Musa was permitted to wear a gold ring, and had a statue erected to him by public subscription near that of Æsculapius. Horace mentions the practice of Musa (Epist. lib. i, 15, 3). Bathing, like many other of the customs of the Romans, from being a natural and healthy recreation, soon became a luxury, which, instead of invigorating, only tended to enervate the body. Thus, during the time when the Romans were under the kings, the waters of the muddy Tiber supplied the people; during the republic, public baths were erected, and private individuals enjoyed the *balneolum*, as Seneca called it, in contradistinction to the more luxurious *balnearium*, with which the villas of the gentry of his own time were fitted up. The too frequent use of the bath was always considered effeminate. The Greeks used the *cold* bath first, and then the *hot* or the *dry air* room. Galen* prescribed the hot air room first, then the tepid or hot water; after this the cold, and then a good rubbing.

* De Meth. Med. vol. x, p. 708, 709, ed. Kühn. Celsus, λ, μ.

Celsus advised the bather to sweat first with his clothes on in the tepid chamber, then to go to the chamber of the *caldarium*, but not to go into the (*solium*) warm bath, but rather to have the tepid, and lastly, to have cold water poured over his head and body; after which he prescribes a good rubbing. The water used by the antient Romans was first of all taken from the Tiber; but subsequently the purest fountains supplied the city with delicious water, conveyed by enormous aqueducts for many miles. These structures, raised at an enormous expense, and sustained by large revenues, at once prove the thorough appreciation of this great people of the hygienic value of personal cleanliness. Linen apparel and more complete covering of the body have done away with a great deal of the actual necessity of the bath, and, comparatively, has made us here in England almost forget the use of it. Where is there a community, five out of every thousand of whom bathe themselves thoroughly as the Romans did, even once a month? In the summer, perhaps, the average number may rise a little; but the proportion of bathers to those who rejoice in the accumulated crust of years are few indeed. The value of having the skin free from dirt need not be here insisted on, for every one acknowledges the fact, although so few are there that carry the practice out in their own persons. We recollect the case of a youth of 18 years, who had never been washed in all his life, *not even at his birth*, on the evidence of his own grandmother, who delivered her daughter, and confessed to this filthy sin of omission; the lad was suffering from acute pneumonia. We immediately set these women to scrub him all over with brush, soap, and flannel; an extraordinary diaphoresis followed this effectual ablution, and the lad was cured without any other means, except an *internal* purgative.

*The Medicinal Baths* of the Romans were principally those of the Albulæ Aquæ, whose waters were often brought to Rome for use. They were about 80° Fahr., and derived their name in all probability from their whiteness. Martial mentions them as smoking; in fact, the sulphurous vapour arises from the lake in such quantities as to characterise the scenery. Thermæ were built in the neighbourhood, the remains of which are still to be traced. Galen considered them efficacious in certain cases of ulcers (skin diseases?), as well as in fluxes, which they arrested in consequence of their astringent properties. We have yet to consider the regulations for the cleanliness and ventilation of their houses and streets, drainage, windows, sleeping apartments, height and aspect of their dwellings, and number of houses and inhabitants.

*Alfred Haviland.*

